# No differences in cardiovascular autonomic responses to mental stress in chronic fatigue syndrome adolescents as compared to healthy controls

**DOI:** 10.1186/1751-0759-4-22

**Published:** 2010-12-14

**Authors:** Caroline Egge, Vegard Bruun Wyller

**Affiliations:** 1 Faculty of Medicine, University of Tromsø, Norway; 2 Division of Pediatrics, Oslo University Hospital Rikshospitalet, Oslo, Norway

## Abstract

Chronic fatigue syndrome (CFS) is a disabling disease with unknown etiology. There is accumulating evidence of altered cardiovascular autonomic responses to different somatic stressors, in particular orthostatic stress, whereas autonomic responses to mental stress remain to be investigated. In this study, we explored cardiovascular autonomic responses to a simple mental stress test in CFS patients and healthy controls.

A consecutive sample of 13 patients with CFS, aged 12 to 18 years, and a volunteer sample of 53 healthy control subjects of equal age and gender distribution were included. Blood pressure, heart rate and acral skin blood flow were continuously recorded during an arithmetic exercise.

At baseline, heart rate was significantly higher among CFS patients than controls (p = 0.02). During the arithmetic exercise, however, there were no significant differences in the responses between the two groups.

In conclusion, CFS patients have unaltered autonomic responses to simple mental stress as compared to healthy control subjects.

## 

Chronic fatigue syndrome (CFS) is a disabling disease with yet unknown aetiology [1]. However, recent research has provided strong evidence of cardiovascular autonomic disturbances during orthostatic stress [2,3], isometric exercise [2] and cooling [4]. Cardiovascular autonomic responses to mental stressors have scarcely been explored in this patient group, despite several reports of attention problems and other specific cognitive impairments [5]. However, in a previous study from our own laboratory, no significant associations were found between autonomic responses during orthostatic stress and degree of psychosocial load [6]. Thus, altered autonomic reactivity in CFS patients might be less related to mental processes than previously thought.

The aim of this study was to explore cardiovascular autonomic responses to an isolated mental stress test in adolescents with CFS. We hypothesized no significant differences in the responses among CFS patients as compared to healthy controls.

Patients with CFS aged 12 to 18 years were consecutively recruited from the outpatient clinic at the Division of Pediatrics, Oslo University Hospital Rikshospitalet, Norway, serving as a national referral center for children and adolescents with unexplained chronic fatigue.

Different case definitions of CFS exist. This study adhered to all of the main criteria in the definition from the Centers for Disease Control and Prevention (CDC) [7]. Specifically, we required 6 months of chronic or relapsing fatigue, severely affecting daily activities; the fatigue should not be explained by any concurrent condition, it should be new or definite in onset, it should not be related to ongoing exertions, and it should not be alleviated by rest. In addition, according to the CDC definition, the patients are also required to report 4 of 8 specific accompanying symptoms. However, recent evidence raises serious concerns about this part of the definition [8], and accompanying symptoms were not required in this study.

Healthy control subjects aged 12 to 18 years volunteered from local schools. The recruiting procedure assured an equal distribution of age and gender among patients and control subjects. A high number of participants in both groups would have yielded best statistical power. However, because control subjects were far easier to recruit than patients with CFS, we aimed at a 4:1 relation. Subjects having a chronic disease (such as allergy, skin diseases, vascular diseases, or diabetes) or using drugs (including contraceptive pills) on a regular basis were excluded.

One week before the experiments, all of the participants were instructed not to drink beverages containing alcohol or caffeine, not to take any drugs, and not to use tobacco products. On the day of the experiments, they were instructed to have fasted overnight.

Written informed consent was obtained from all of the participants and their parents. The study was conducted in accordance with the Helsinki declarations, and was approved by the Regional Committee for Ethics in Medical Research.

During the experiments, subjects were lightly dressed and lay supine in a climatic chamber with an ambient temperature of 26°C, assuring that they were within thermoneutral zone. The left hand was immersed in a stirred thermostat-controlled water bath (CB 29-20e; Heto-Holtan, Åbyhøj, Denmark) having a temperature of 35°C, which corresponds to thermoneutrality in water. This experimental set-up was required for subsequent tests of thermoregulatory autonomic control, as has been reported elsewhere [4].

After 30 minutes of acclimatization, but prior to the thermoregulatory test, the subjects were asked to subtract seven from hundred, and then repeat the subtraction of seven down to zero. They were instructed to perform the arithmetic in a steady, concentrated way, avoiding "rushing" as well as "dwelling", and to continue subtraction despite concerns about the correctness of the answers. Care was taken to ensure that the subjects understood the instructions. Erroneous answers were not corrected by the investigator. This simple, standardized arithmetic exercise is a validated test of attention within the Mini Mental State Examination [9]; previously, very similar tests have been shown to provoke strong autonomic responses [10].

Continuous recordings of acral skin laser flux, which is a measure of acral skin blood flow (ASBF), were obtained by a laser-Doppler instrument (DRT4; Moore Instruments, Milway, Devon, United Kingdom) [11]. Instantaneous heart rate (HR) was obtained from the R-R interval of the electrocardiogram. Photoplethysmography on the right third finger was used to obtain a non-invasive, continuous recording of arterial blood pressure (2300 Finapres; Ohmeda, Madison, WI). This method correlates satisfactorily with invasive pressure measurements [12], and has also been validated in adolescents and children. All signals were transferred online to a recording computer running a program for real-time data acquisition (developed by Morten Eriksen, Department of Physiology, University of Oslo, Oslo, Norway). Beat-to-beat mean blood pressure (MBP) was calculated by numerical integration of the recorded instantaneous blood pressure. The other physiologic variables were also converted to beat-to-beat records.

Data were exported to Microsoft Excel (Microsoft, Redmond, WA) for further calculations. We defined three epochs of interest, each of 10 seconds' length: Baseline (from 55 to 45 sec prior to mental exercise), Early test (from 15 to 25 sec into the exercise), and Late test (the last 10 sec of the exercise), and computed Delta 1 (Early test - Baseline) and Delta 2 (Late test - Baseline).

SPSS statistical software (SPSS Inc, Chicago, IL) was used for statistical analyses. On the basis of inspection of plots, most variables were appraised not to follow a normal distribution. The data are therefore reported as medians with nonparametric confidence intervals (CI) for all variables. The non-parametric *Mann-Witney U's*-test (two-sided) was used to compare results among patients and controls. As the research question did not concern within-group changes, no one-sample tests were carried out. A p < 0.05 was considered statistically significant.

A total of 13 CFS patients and 53 healthy controls were included in the study (Table 1). The two groups were comparable regarding sex, age, weight, and height. All were of Caucasian ethnicity, except one control. CFS patients reported severe functional impairments; they were physically inactive, did not participate in leisure activities and had a high level of school absence. However, no one was permanently bedridden.

**Table 1:** Subject characteristics

	*CFS patients*	*Healthy controls*
Total number	13	53
Female gender	8 (61.5%)	32 (60.4%)
Mean age, years (SD)	15.6 (1.6)	15.6 (1.8)
Mean weight, kg (SD)	61 (16)	62 (11)
Mean height, cm (SD)	173 (10)	172 (10)

At baseline, HR was significantly higher in CFS patients than controls (p = 0.02) (Table 2). No other significant differences were detected. As compared to baseline values, blood pressure and HR increased and ASBF decreased in both groups during the test. During the first part of mental exercise, HR, MBP and DBP tended to increase less in patients than in controls; however, the differences between the two groups did not reach statistical significance. At Late test, the changes from baseline were almost identical among patients and controls for most variables. Of note, for all variables, the confidence intervals of the medians were in the same range both at Delta 1 (Early test - Baseline) and Delta 2 (Late test - Baseline). The time used to complete the test was approximately equal in the two groups; median (CI) were 81 sec (46 to 102) among CFS patients and 67 sec (61 to 76) among healthy controls, respectively.

**Table 2:** Cardiovascular variables during mental exercise

	*Baseline*		*Delta 1(Early test - Baseline)*		*Delta 2 (Late test - Baseline)*	
			
	CFS patients (n = 13)	Healthy controls (n = 53)	CFS patients (n = 13)	Healthy controls (n = 53)	CFS patients (n = 13)	Healthy controls (n = 53)
ASBF Right (a.u.)	256	304	-51	-43	-76	-39
	(127 to 321)	(222 to 354)	(-165 to -2)	(-88 to -14)	(-194 to -37.2)	(-81 to -8)
ASBF Left (a.u.)	278	300	-42	-68	-104	-29
	(139 to 290)	(202 to 349)	(-155 to -12)	(-107 to -25)	(-183 to -32)	(-80 to -4)
MAP (mm Hg)	87.1	78.6	4.3	8.1	11.1	12.0
	(76.7 to 89.6)	(75.2 to 81.4)	(2.4 to 8.3)	(6.7 to 10.5)	(9.4 to 12.1)	(10.4 to 14.4)
SBP (mm Hg)	132.3	127.0	10.8	10.9	12.9	14.8
	(117.3 to 135.5)	(118.3 to 130.6)	(1.3 to 14.0)	(4.4 to 14.9)	(11.6 to 19.9)	(10.4 to 20.9)
DBP (mm Hg)	66.2	60.6	3.8	7.1	11.2	11.2
	(58.6-75.2)	(58.4 to 64.9)	(-0.9 to 8.3)	(4.3 to 9.4)	(9.2 to 11.6)	(10.1 to 12.6)
HR (beats/min)	81.4*	73.5	9.4	17.1	7.8	10.3
	(71.9 to 89.2)	(68.4 to 76.6)	(3.7 to 18.9)	(12.7 to 19.6)	(4.1 to 19.0)	(7.4 to 13.0)

* Significant higher than controls (p < 0.02), Mann-Whitney U's test (two-sided). ASBF = Acral skin blood flow (right and left finger), a.u. = arbitrary units, MAP = Mean arterial pressure, SBP = Systolic blood pressure, DBP = Diastolic blood pressure, HR = Heart rate.

Median (Confidence intervals).

The main result of this study is that CFS patients and healthy controls have a similar cardiovascular autonomic response to a mental stress test, confirming our hypothesis. This result suggests that abnormal cardiovascular autonomic responses in CFS are not an unspecific reaction towards all kinds of challenges, but more restricted to certain somatic stressors.

In previous studies of CFS patients, both hyper-reactivity and hypo-reactivity have been reported [13]. According to La Manca and co-workers, this discrepancy might be explained by qualitative and quantitative differences in the stressors applied. More specifically, a relatively light mental exercise, as in our study, might be insufficient in provoking different autonomic responses among patients and controls. Furthermore, during the exercise, the subjects were shielded from other stressors, such as noise, light, cold and the physical effort of being upright. The response to a heavier mental task, as well as the response to combined mental and physical stressors, should be explored in further studies.

We had a relatively small group of patients, weakening the statistical power. Thus, we cannot rule out the possibility of type 2-errors. However, the changes in all cardiovascular variables during the experiment were very similar in CFS patients and controls, as judged both from the medians and from the confidence intervals of the medians. Furthermore, previous experiments on the same group of CFS and control subjects did document significant differences in cardiovascular autonomic responses to orthostatic stress, isometric exercise and cooling [2,4]. Thus, in this study, we have reason to believe that the number of participants included as well as the recording methods applied does possess the necessary power and sensitivity to detect differences between the two groups if they are present.

Although not the scope of this study, it should be noted that the baseline HR was significantly higher among CFS patients as compared to controls. This is in line with previous reports from our laboratory [2,4], suggesting a sympathetic predominance of heart rate control at rest. However, different autonomic control at baseline does not imply different responses to a certain stressor; rather, the autonomic responses are tailored to a specific event, being determined by genetic factors as well as conscious and unconscious mental processes [14].

Variability analyses, being a useful and widespread method of studying autonomic cardiovascular control, were not applied in this study; such analyses require stationarity of the biosignals, which could not be assumed in our experiment [15]. Furthermore, apart from time to complete the test, we did not record if there were any differences in *performance *of the test, which might be a confounding factor. Another study limitation is the different activity-levels among patients and controls which might explain the baseline-differences in HR; however, none of the patients was permanently bed-ridden.

## List of abbreviations

ASBF: Acral skin blood flow; CDC: Centers for Disease Control and Prevention; CFS: Chronic fatigue syndrome; DBP: Diastolic blood pressure; HR: Heart rate; MBP: Mean blood pressure; SBP: Systolic blood pressure.

## Declaration of competing interests

The authors declare that they have no competing interests.

## Authors' contributions

CE performed the data analyses and drafted the manuscript. VBW conceived of the study, carried out the experiments, participated in data analyses and helped to draft the manuscript. Both authors have read and approved the final version of the manuscript.
